# Depression and Pesticide Exposures among Private Pesticide Applicators Enrolled in the Agricultural Health Study

**DOI:** 10.1289/ehp.11091

**Published:** 2008-09-09

**Authors:** Cheryl L. Beseler, Lorann Stallones, Jane A. Hoppin, Michael C.R. Alavanja, Aaron Blair, Thomas Keefe, Freya Kamel

**Affiliations:** 1 Colorado Injury Control Research Center, Department of Psychology, Colorado State University, Fort Collins, Colorado, USA; 2 Epidemiology Department, College of Public Health; 3 Department of Environmental, Agricultural and Occupational Health, University of Nebraska Medical Center, Omaha, Nebraska, USA; 4 Epidemiology Branch, National Institutes of Environmental Health Sciences, National Institutes of Health, Department of Health and Human Services, Research Triangle Park, North Carolina, USA; 5 Occupational and Environmental Epidemiology Branch, Division of Cancer Epidemiology and Genetics, National Cancer Institute, National Institutes of Health, Department of Health and Human Services, Rockville, Maryland, USA; 6 Department of Environmental and Radiological Health Sciences, Colorado State University, Fort Collins, Colorado, USA

**Keywords:** cumulative exposure, depression, farm applicators, pesticides, pesticide poisoning

## Abstract

**Background:**

We evaluated the relationship between diagnosed depression and pesticide exposure using information from private pesticide applicators enrolled in the Agricultural Health Study between 1993 and 1997 in Iowa and North Carolina.

**Methods:**

There were 534 cases who self-reported a physician-diagnosed depression and 17,051 controls who reported never having been diagnosed with depression and did not feel depressed more than once a week in the past year. Lifetime pesticide exposure was categorized in three mutually exclusive groups: low (< 226 days, the reference group), intermediate (226–752 days), and high (> 752 days). Two additional measures represented acute high-intensity pesticide exposures: an unusually high pesticide exposure event (HPEE) and physician-diagnosed pesticide poisoning. Logistic regression analyses were performed relating pesticide exposure to depression.

**Results:**

After adjusting for state, age, education, marital status, doctor visits, alcohol use, smoking, solvent exposure, not currently having crops or animals, and ever working a job off the farm, pesticide poisoning was more strongly associated with depression [odds ratio (OR) = 2.57; 95% confidence interval (CI), 1.74–3.79] than intermediate (OR = 1.07; 95% CI, 0.87–1.31) or high (OR = 1.11; 95% CI, 0.87–1.42) cumulative exposure or an HPEE (OR = 1.65; 95% CI, 1.33–2.05). In analysis of a subgroup without a history of acute poisoning, high cumulative exposure was significantly associated with depression (OR = 1.54; 95% CI, 1.16–2.04).

**Conclusion:**

These findings suggest that both acute high-intensity and cumulative pesticide exposure may contribute to depression in pesticide applicators. Our study is unique in reporting that depression is also associated with chronic pesticide exposure in the absence of a physician-diagnosed poisoning.

Recent reviews have noted neurologic sequelae of pesticide exposure among farmers (Jamal et al. 2002a; Kamel and Hoppin 2004), and in the past decade strong associations have been reported between high-level occupational exposure to pesticides and an increased risk of depression (Amr et al. 1997; Farahat et al. 2003; Stephens et al. 1995). In an Egyptian population, the frequency of depressive neurosis was significantly higher in male pesticide formulators with at least 15 years of pesticide exposure compared with matched controls (Amr et al. 1997). In the same study, severe depression scores from the General Health Questionnaire (GHQ) were significantly higher in male pesticide applicators than in their non-exposed controls matched on age, socioeconomic status, and educational levels. The exposures considered were primarily carbamates, pyrethroids, and organophosphates (OPs) applied to cotton fields; although pesticide poisoning was not reported, the investigators noted that safety measures were poor in this study population (Amr et al. 1997). Another Egyptian study of 52 male agricultural engineers, pesticide mixers, and mechanics who worked in pesticide application showed a higher neuroticism score compared with 50 demographically similar clerks and administrators (Farahat et al. 2003). Vulnerability to psychiatric disorders, as determined by the GHQ, was found to be 50% greater in 146 OP-exposed sheep farmers compared with 143 quarry worker controls in the United Kingdom (Stephens et al. 1995). However, a study of 57 male tree-fruit farmers exposed to OPs showed no significant difference in depression rates when compared with 42 age-matched, unexposed cranberry/blueberry growers (a farming control group) or hardware store owners (Fielder et al. 1997).

A number of studies have shown long-term depression as a sequela of an acute pesticide poisoning (Beseler et al. 2006; Reidy et al. 1992; Rosenstock et al. 1991; Savage et al. 1988; Stallones and Beseler 2002). Having experienced a pesticide-related illness was significantly associated with depression measured using the Center for Epidemiologic Studies Depression Scale (CES-D) in 761 Colorado farm residents (Stallones and Beseler 2002). In 52 tobacco farmers in southern Brazil who were applying OPs (13 reporting a history of poisoning), the number of individuals with depression or anxiety disorders was significantly higher than expected but declined after 3 months without OP exposure (Salvi et al. 2003). The level and duration of pesticide exposure resulting in depression remain unclear. Although the above studies suggest a high-level exposure is needed, mechanisms have been proposed that could explain an association with chronic, low-dose toxicity (Brown and Brix 1998; Browne et al. 2006; Ray and Richards 2001). Anxiety and depression have been observed in the course of conducting extensive neurologic examinations but only recently have been the focus of hypothesis-driven research. For example, feeling depressed, indifferent, or withdrawn during the last 12 months, one of many self-reported neurologic symptoms examined, was associated with cumulative pesticide exposure among licensed pesticide applicators in the Agricultural Health Study (AHS) (Kamel et al. 2005).

The purpose of the present study was to evaluate the relationship between pesticide exposure and physician-diagnosed depression in a cohort of farmer applicators enrolled in the AHS.

## Materials and Methods

The AHS cohort includes 89,658 individuals (4,916 commercial applicators, 52,395 private applicators, and 32,347 spouses of private applicators) who were recruited in Iowa and North Carolina from 1993 through 1997 and who are being followed to assess a variety of health outcomes (Blair et al. 2005). The present analysis was restricted to private applicators, who were primarily farmers. Commercial pesticide applicators were excluded from the study because they have patterns of exposure different from those of farmer applicators (Alavanja et al. 1999; Arbuckle et al. 2002); female spouses were considered in a previous publication (Beseler et al. 2006). The current study is a cross-sectional analysis of baseline data from the ongoing cohort study.

All applicators completed a questionnaire at enrollment, and 44% (22,916) completed a supplemental questionnaire at home. Together, the questionnaires provided detailed information on physician-diagnosed depression, pesticide use, and covariates, including race, ethnicity, education, marital status, medical conditions, alcohol use, and smoking habits (Alavanja et al. 1996). Copies of the questionnaires are available online (AHS 2008).

Information on physician-diagnosed depression and physician-diagnosed pesticide poisoning was requested only in the supplemental questionnaire, so we included only those applicators who completed the latter in the present analysis. Although generally similar to the entire AHS population, the non-responders, who were significantly younger than those who responded to the questionnaire, spent more time mixing and applying pesticides than the responders and had slightly greater cumulative exposure (Tarone et al. 1997). Of the 22,916 applicators who returned the supplemental questionnaire, 882 applicators with missing depression information, 96 with previous lead or solvent poisoning, 28 who were < 18 years of age including one whose age was unknown, and 556 women were excluded from the study. Lead poisoning and solvent poisoning are strongly associated with neurologic effects (Walker 2000), and depression in men is different from depression in women (Kornstein et al. 1996).

Cases were defined as farmer applicators who responded “yes” to the question, “Has a doctor ever told you that you had (been diagnosed with) depression requiring medication or shock therapy?” Those responding “yes” were asked about their age at diagnosis in four categories (< 20, 20–39, 40–59, > 59 years). Controls were farmer applicators who had not been diagnosed with depression. To reduce potential misclassification, 848 individuals who reported feeling depressed, indifferent, or withdrawn once a week or more during the year prior to enrollment, but had not been diagnosed with depression, were excluded from the control group. This left a final study population of 17,585, with 534 cases of diagnosed depression and 17,051 controls who had complete information for analyses. In a supplemental analysis, we included the 848 individuals with depressive symptoms at least once a week in the control group to determine the impact of the exclusion on our results.

Cumulative exposure to any pesticide was categorized in three groups, based on the distribution of lifetime application days. The latter were calculated as the average number of days per year the applicator mixed or applied any pesticide (asked in six categories) multiplied by the total number of years of pesticide use (asked in seven categories), using the midpoints of the categories. The low-exposure group (reference group) had used pesticides < 226 days (the median); the intermediate-exposure group reported 226–752 days of use (the median to the 90th percentile); and the high-exposure group reported > 752 days of use (≥ 90th percentile). Two additional variables, identifying applicators who had ever experienced an unusually high pesticide exposure event (HPEE) or physician-diagnosed pesticide poisoning, were created, regardless of lifetime application days of use. Those who answered “yes” to the question “Have you ever had an incident or experience while using any type of pesticide which caused you unusually high personal exposure?” were categorized as having had an HPEE. Those who answered “yes” to “Has a doctor ever told you that you had (been diagnosed with) pesticide poisoning?” were classified as having had a pesticide poisoning. These variables were not mutually exclusive, that is, those with an HPEE would also have a value for lifetime days of exposure and an indicator for the presence or absence of a history of pesticide poisoning, and those with a history of pesticide poisoning would have a value for lifetime days of exposure and an indicator for the presence or absence of an HPEE.

The questionnaires also collected detailed information on the lifetime use of 50 pesticides: 22 on the enrollment questionnaire and 28 on the supplemental questionnaire. Functional pesticide groups were created by combining data for specific chemicals, including 18 herbicides, 22 insecticides, 6 fungicides, and 4 fumigants. Three groups of insecticides were analyzed: OPs (malathion, parathion, diazinon, phorate, coumaphos, dichlorvos, chlorpyrifos, fonofos, terbufos, and trichlorfon); carbamates (carbaryl, carbofuran, and aldicarb); and organochlorines (aldrin, chlordane, dieldrin, DDT, heptachlor, and toxaphene).

We selected covariates evaluated in univariate logistic regression models based on previous studies of pesticides or depression and included age, state of residence, high school education, race (white, other), Hispanic ethnicity, marital status, smoking behavior (Breslau et al. 1998), alcohol use, frequency of visits to a doctor in the past year, farms with no crops or animals, working a job off the farm, number of years lived or worked on a farm, and use of personal protective equipment (PPE) (Scarth et al. 2000; Stallones et al. 1995). Those who were legally married or living as married were classified as married and those who were divorced, widowed, or never married were considered unmarried. Age was categorized into four levels based roughly on quartiles, because those who were younger or older were less likely to be depressed than those in the middle years. Alcohol use in the past year was categorized as never, less than one drink per week, one to four drinks per week, and daily or almost daily. We used one to four drinks per week as the reference group because individuals with this level of alcohol use had the lowest risk of depression. Those who reported never using PPE when handling pesticides were compared with those who reported using PPE. In addition, individual types of PPE were analyzed separately including fabric/leather gloves, chemically resistant gloves, face shields, respirators, and disposable clothing. Solvent information from a question asking about exposures from the nonfarm job held the longest was included in the multivariable models to adjust for the association of solvents with neurologic conditions (Walker 2000). On- and off-farm exposure to metals, such as lead solder, welding and electroplating fumes, lead, mercury, cadmium, and other metals, as well as additional exposures to gasoline and other solvents, were also evaluated as potential confounders of the relationship between pesticide exposure and physician-diagnosed depression.

We used univariate and multivariable models to evaluate associations of depression with cumulative exposure to any pesticide or with functional pesticide groups. Multivariable models were adjusted for potential confounders that were significantly associated with both depression in the univariate analyses and also associated with the three cumulative categories of exposure to any pesticide, an HPEE, or pesticide poisoning; the models excluded additional pesticide exposure variables to avoid bias due to overcontrol. Covariates included in the final models were state of residence, marital status, age, off-farm solvent exposure, alcohol use, smoking behavior, education, working a job off the farm, doctor visits, and having no crops or animals on the farm.

To separate the effects of cumulative lifetime exposure from those of high exposure, we ran three multivariable models:

- Model 1 included all subjects.
- Model 2 excluded those who reported an HPEE.
- Model 3 excluded those who reported a pesticide poisoning.
- Model 4 excluded those who reported an HPEE or a pesticide poisoning.

Although state of residence was not significant in univariate models, it was included in multivariable models to adjust for economic differences resulting from farm size, products, and differences in farming practices between states. For the entire sample, we ran a single multivariable model that included the three level cumulative exposure variable and two separate variables for HPEE and pesticide poisoning. We also conducted the same analyses separately by state of residence to determine whether associations were found in both states and to identify any differences between the states.

Results are reported as odds ratios (ORs) with 95% confidence intervals (CIs).

All analyses were conducted using SAS version 9.1 (SAS Institute, Cary, NC, USA). We used the 2 August 2002 release of the AHS phase I dataset (Prerelease 0208) for all analyses. This work was conducted with approval from the Institutional Review Boards of Colorado State University, the National Institute of Environmental Health Sciences, and the National Cancer Institute. All study participants gave written informed consent prior to the study.

## Results

In this study population, 3.0% reported a physician-diagnosed depression; prevalence was similar in North Carolina (2.8%) and Iowa (3.1%). We compared demographic characteristics between cases and controls in those with complete data for all covariates (Table 1). Race and ethnicity did not differ between cases and controls, but there were very few nonwhites or Hispanics in the study population. Cases were more likely than controls to be older, be unmarried, have more visits to the doctor, be a past smoker, consume alcohol less frequently, have ever worked off the farm, and presently live on a farm with no crops or animals, but there were no differences between cases and controls in the lifetime number of years lived or worked on a farm (Table 1). The use of PPE for pesticide application did not differ between cases and controls (Table 1). Further analysis by type of PPE, including fabric/leather work gloves, chemically resistant gloves, gas mask, face shield, and disposable clothing, showed that only wearing chemically resistant gloves was protective against being depressed (OR = 0.79; 95% CI, 0.64–0.97). Cases were more likely to have had exposure to solvents and heavy metals in the nonfarm job that they held the longest (Table 1). Exposures to other solvents (gasoline and cleaning solvents) from both on- and off-farm exposures were not significantly associated with depression. No significant association was observed between diagnosed depression and lifetime days of pesticide exposure in univariate analysis; however, cases were more likely to have spent a greater number of years mixing and applying pesticides. Significantly increased odds of depression were observed for individuals who reported an HPEE and those with a history of pesticide poisoning. The median lifetime days of pesticide exposure was significantly higher in both those who reported an HPEE and those who reported a history of pesticide poisoning (370 days) compared with those who did not report either of these events (225 days) (*p* < 0.0001). No significant interactions were observed between any of the demographic variables and any of the exposure levels.

Table 2 presents multivariable logistic regression of factors showing a significant relationship with depression in the univariate analyses shown in Table 1. State of residence was also included, although it was not significant in the univariate analysis, because farmers living in Iowa were more likely to be diagnosed with depression than farmers in North Carolina in the multivariable model (Table 2). After excluding those with an HPEE, pesticide poisoning, or HPEE and/or poisoning, the highest level of lifetime days (> 752) showed a statistically significant relationship to diagnosed depression.

Using the same approach as in Table 2, the results were nearly identical when those who reported experiencing depressive symptoms in the past year were left in the control group in multivariable models (226–752 days: OR = 1.06; 95% CI, 0.86–1.30; > 752 days: OR = 1.10; 95% CI, 0.86–1.41; HPEE: OR = 1.61; 95% CI, 1.30–2.00; poisoning: OR = 2.48; 95% CI, 1.69–3.66).

Table 3 shows results with all exposure variables stratified by state of residence. With HPEE and pesticide poisoning in the model, the highest cumulative exposure was not significantly associated with depression. The relationship of depression to history of pesticide poisoning was slightly stronger for North Carolina than for Iowa farmers, whereas the association with HPEE was weaker. In both states, the ORs were greater for pesticide poisoning than for cumulative pesticide exposure or an HPEE.

Table 4 shows results for functional and chemical pesticide groups. At least 98% of cases and controls reported ever using herbicides, and greater than 93% reported using crop or animal insecticides. Ever having used herbicides showed a strong association with diagnosed depression in adjusted models, but the CI included 1.0 (Table 4). Ever having used insecticides, OPs, or organochlorines was significantly associated with diagnosed depression in both unadjusted and adjusted models (Table 4). In subsequent analyses of cumulative days of use, a dose–response relationship was not observed for any group (data not shown). Stepwise logistic regression selected ever use of OPs and ever use of organochlorines to be significant predictors of depression in unadjusted models (OR = 1.56; 95% CI, 1.12–2.19; OR = 1.34; 95% CI, 1.13–1.60, respectively) and in adjusted models (OR = 1.61; 95% CI, 1.14–2.28; OR = 1.24, 95% CI, 1.03–1.50, respectively).

## Discussion

In the present study, depression was associated with a history of pesticide poisoning or an HPEE after adjusting for important covariates. In addition, after excluding subjects with an HPEE or poisoning, high cumulative pesticide exposure was significantly associated with diagnosed depression. We observed similar results when subjects with depressive symptoms were included in the control group. These findings extend previous reports where symptoms of anxiety and depression were reported in pesticide-poisoned individuals (Reidy et al. 1992; Savage et al. 1988; Stokes et al. 1995). Acutely exposed migrant farm workers (*n* = 21) with documented cholinesterase inhibition reported feeling depressed and anxious significantly more often than unexposed controls (*n* = 11) matched on age, sex, education, socioeconomic status, and ethnicity (Reidy et al. 1992). Savage et al. (1988) and Steenland et al. (1994) found significant alterations in mood in subjects who had experienced a pesticide poisoning from 3 to > 10 years prior to evaluation. The present study is unique in reporting that depression is also associated with chronic pesticide exposure in the absence of a physician-diagnosed poisoning.

In the present study, ever having used insecticides, OPs, or organochlorines was significantly associated with diagnosed depression. Long-term exposure to DDT has been associated with an increase in psychiatric symptoms (van Wendel de Joode et al. 2001). DDT alone was not significantly associated with depression in this farming population in univariate (OR = 1.17; 95% CI, 0.96–1.42) or adjusted models (OR = 1.09; 95% CI, 0.87–1.37). Carbamates were not associated with depression, indicating that the rapid reactivation of acetylcholinesterase (ACHE) after carbamate inhibition may reduce the risk of depression, or an altogether different mechanism may explain the association of OPs and depression in previous studies and in the current one.

Genetic susceptibility may play a role in the association of depression with pesticide exposure. Those with a personal or family history of affective disorders may be at increased risk of depression when exposed to ACHE-inhibiting OPs (Janowsky et al. 1994). The increased risk in those with long-term pesticide exposure might be due to genetic vulnerability to pesticide effects resulting from a paraoxonase polymorphism (Browne et al. 2006; Cherry et al. 2002; Costa et al. 2003) or other genetic variant.

Several mechanisms have been proposed for subthreshold exposure resulting in delayed, long-term depression (Browne et al. 2006; Bryk et al. 2005; Kaufer et al. 1998; Shapira et al. 2000). Inhibition of ACHE results in neuronal hyperexcitability and increased production of an alternative ACHE mRNA product known as ACHE-R, which cannot form multimers. Increased ACHE-R causes changes in gene expression via c-*fos* and subsequent changes in protein expression, leading to a reduction in acetylcholine and a decrease in choline acetyltransferase and vesicular acetylcholine transporter proteins (Kaufer et al. 1998). All of these changes represent the cholinergic system trying to react to stress and trying to repair the imbalance due to over-stimulation (Kaufer et al. 1998). Although useful in the short term, over time these changes can be deleterious and may lead to psychiatric problems. Additionally, polymorphisms in the ACHE promoter may cause some individuals to be more sensitive to ACHE-inhibiting pesticides (Shapira et al. 2000), and polymorphisms in both paraoxonase and ACHE may interact to further increase susceptibility (Bryk et al. 2005).

An alternative explanation for the positive findings in the high cumulative pesticide exposure category is that applicators in this category were more likely to have had an unreported HPEE or poisoning. The reporting of lifetime pesticide use in the AHS has been evaluated and shown to be reliable (Blair et al. 2002); however, the reliability of reporting an HPEE or a pesticide poisoning is unknown. Although use of mutually exclusive categories for analysis suggests that high cumulative exposure was associated with depression independent of HPEE or poisoning, the reverse is not necessarily true. Those reporting an HPEE or poisoning were also likely to have had high cumulative exposure, and it cannot be determined which of these exposure events led to an increased risk of depression. It is possible that both contribute and that an HPEE or poisoning against a background of high cumulative exposure is associated with increased risk of depression.

Overall, the prevalence of physician-diagnosed depression (3.0%) was relatively low in this farming population compared with community surveys using the CES-D with a score of ≥16 as an indicator of clinical depression. Using the CES-D, male farm residents showed a prevalence of depression of 7.9% in Colorado (Stallones et al. 1995), 12.2% in Iowa (Scarth et al. 2000), and 20% in a farming community in Tennessee (Linn and Husaini 1987). The difference between diagnosed depression and depression determined from a survey instrument is a function of propensity to seek treatment, access to health care in the rural community, and the training of physicians to ask about depressive symptoms, as well as the actual prevalence of depression. It could be that AHS participants are not likely to seek treatment for mental health conditions, as was reported by Linn and Husaini (1987), where only 5.3% of farmers who were depressed using the CES-D sought medical treatment (Linn and Husaini 1987). Even if individuals recognize depression and seek treatment, they may not report their condition on a questionnaire. Differences among communities may also be related to a number of economic conditions, including farm size and products produced.

Those visiting a doctor at least two times during the past year showed twice the odds of being diagnosed with depression compared with those with only one visit. This agrees with a previous study showing that a greater number of visits to a doctor increased the chances of being diagnosed with depression (Bertakis et al. 2001). Conversely, those who are depressed may visit a doctor more often. To examine importance of this reciprocal relationship, we stratified the analysis by the number of doctor visits and found that although a history of pesticide poisoning was 2-fold higher in those without a doctor visit in the past year compared with those with one or more visits, pesticide poisoning was the most important predictor of depression in both strata of doctor visits. The results were only slightly greater in multivariable models that did not adjust for number of doctor visits in the past year (medium cumulative days: OR = 1.08; 95% CI, 0.88– 1.32; high cumulative days: OR = 1.15; 95% CI, 0.90–1.48; HPEE: OR = 1.81; 95% CI, 1.46–2.24; pesticide poisoning: OR = 2.73; 95% CI, 1.86–4.02). A limitation of this analysis is that doctor visits in the 12 months prior to completing the supplemental questionnaire may not represent doctor visits during the time the depression diagnosis was made. Because of the lack of adequate temporal information, it can only be concluded that the relationship between a history of pesticide poisoning and diagnosed depression was not a function of the number of doctor visits in the year preceding enrollment into the AHS.

The strengths of the present study include the large number of cases of self-reported physician-diagnosed depression, a detailed questionnaire containing information on pesticide use history and other risk factors for depression, a gradation of exposure levels, and controls drawn from the same population as cases, which minimizes potential confounding.

A limitation is that information on diagnosed depression and pesticide exposure was collected cross-sectionally, and both were self-reported. Additionally, age at diagnosis of depression and age at pesticide poisoning were asked in 20-year age categories, and no specific dates were available for either diagnosis. It is possible that depression could increase the probability of an HPEE or pesticide poisoning. However, of the 33 individuals who had both a diagnosis of pesticide poisoning and depression and provided information on the age category when these diagnoses occurred, only one individual reported depression prior to pesticide poisoning.

A further limitation is that data on some potential confounding factors, such as financial information, including income and debt, and indicators of social support, were not available. These factors could be related to stress in the AHS participants, which may increase the probability of having a pesticide poisoning and may also result in depression. It is also possible that those who did not return the supplemental questionnaire were more likely to be depressed than the 44% who did return it. This would lead to an underestimation of the effect sizes of pesticide exposure on depression, especially in those with an HPEE or pesticide poisoning, where the effects appear to be greater.

It is possible that a pesticide poisoning may not result in a diagnosis, either because treatment was not sought or because a physician did not recognize it as a pesticide poisoning. These individuals would have been misclassified with respect to diagnosed poisoning but may have reported the event as an unusually high exposure. These individuals would have fallen into the HPEE category regardless of their cumulative days of exposure. Misclassification of cumulative exposures is also possible. Repeat interviews conducted in Iowa on 4,088 applicators found a high degree of agreement in ever/never use of specific pesticides and application practices, and less agreement for duration, frequency, and decade of first pesticide use (Blair et al. 2002). However, most of the responses were within one category from the response given a year previously (Blair et al. 2002). Collapsing cumulative exposure into three levels most likely minimizes potential misclassification.

## Conclusion

In this study, based on questionnaire data from a well-defined agricultural cohort, high cumulative exposure in the absence of an HPEE or pesticide poisoning, as well as a history of either HPEE or physician-diagnosed pesticide poisoning, was significantly associated with physician-diagnosed depression. The pattern was similar in separate analyses for Iowa and North Carolina.

Physicians should be alert to mood changes in those with a history of applying pesticides. A review of the literature shows that whenever neurologic effects are observed and mood is examined, they tend to be related (Farahat et al. 2003; Jamal et al. 2002b; Stephens et al. 1995). These results suggest that pesticide exposure may contribute to depression in farmer applicators and emphasize the importance of minimizing pesticide exposures. Future work on neurologic effects of pesticide exposure should include measures of affective disorders, including depression and anxiety.

## Figures and Tables

**Table 1:** Demographic, behavioral, medical, and farm characteristics and exposure differences between depression cases (*n* = 534) and controls (*n* = 17,051) in 17,585 Iowa and North Carolina farmer pesticide applicators, 1993–1997.

Characteristic	Cases [no. (%)]	Controls [no. (%)]	OR (95% CI)
State of residence			
Iowa	385 (72.1)	11,865 (69.6)	Reference
North Carolina	149 (27.9)	5,186 (30.4)	0.89 (0.73–1.07)
Age (years)			
< 40	95 (17.8)	4,720 (27.7)	Reference
40–49	164 (30.7)	4,513 (26.5)	1.81 (1.40–2.33)
50–59	163 (30.5)	3,936 (23.1)	2.06 (1.59–2.66)
> 59	112 (21.0)	3,882 (22.8)	1.43 (1.09–1.89)
High school education			
Yes	481 (90.1)	15,735 (92.3)	Reference
No	53 (9.9)	1,316 (7.7)	1.32 (0.99–1.76)
Married/living as married			
Yes	438 (82.0)	14,525 (85.2)	Reference
No	96 (18.0)	2,526 (14.8)	1.26 (1.01–1.58)
Doctor visits past year			
None	81 (15.2)	6,174 (36.2)	Reference
One	131(24.5)	5,431 (31.9)	1.84 (1.39–2.43)
More than one	322 (60.3)	5,446 (31.9)	4.51 (3.52–5.77)
Alcohol use in past year			
Never	198 (37.1)	5,783 (33.9)	1.41 (1.11–1.78)
< 3 per month	190 (35.6)	5,715 (33.5)	1.36 (1.08–1.73)
1–4 per week	110 (20.6)	4,515 (26.5)	Reference
Every/almost every day	36 (6.7)	1,038 (6.1)	1.42 (0.97–2.09)
Cigarette smoking			
Never	255 (47.7)	9,460 (55.5)	Reference
Past	208 (39.0)	5,461 (32.0)	1.41 (1.17–1.70)
Current	71 (13.3)	2,130 (12.5)	1.24 (0.95–1.62)
Ever work a job off the farm			
No	162 (30.3)	6,091 (35.7)	Reference
Yes	372 (69.7)	10,960 (64.3)	1.28 (1.06–1.54)
Farms currently having no crops or animals			
No	524 (98.1)	16,928 (99.3)	Reference
Yes	10 (1.9)	123 (0.7)	2.63 (1.37–5.03)
No. of years lived or worked on a farm			
< 5	9 (1.7)	186 (1.1)	Reference
5–10	8 (1.5)	305 (1.8)	0.54 (0.21–1.43)
11–20	38 (7.2)	1,129 (6.7)	0.70 (0.33–1.46)
21–30	58 (10.9)	2,308 (13.7)	0.52 (0.25–1.07)
> 30	417 (78.7)	12,877 (76.6)	0.67 (0.34–1.32)
PPE			
Used	485 (90.8)	15,651 (91.8)	Reference
Not used	49 (9.2)	1,400 (8.2)	1.13 (0.84–1.52)
Solvent exposure^a^			
No	419 (78.5)	14,206 (83.3)	Reference
Yes	115 (21.5)	2,845 (16.7)	1.37 (1.11–1.69)
Heavy metal exposure^a^			
No	396 (76.2)	13,355 (80.2)	Reference
Yes	124 (23.8)	3,287 (19.8)	1.27 (1.04–1.56)
Days per year mixed/applied			
< 5	88 (16.5)	3,140 (18.4)	Reference
5–9	144 (27.0)	4,262 (25.0)	1.21 (0.92–1.58)
10–19	160 (30.0)	5,309 (31.1)	1.08 (0.83–1.40)
20–39	92 (17.2)	3,084 (18.1)	1.06 (0.79–1.43)
> 39	50 (9.4)	1,256 (7.4)	1.42 (1.00–2.02)
Years mixed/applied			
< 6	33 (6.2)	2,057 (12.1)	Reference
6–10	62 (11.6)	2,403 (14.1)	1.61 (1.05–2.46)
11–20	176 (33.0)	5,621 (33.0)	1.95 (1.34–2.84)
21–30	163 (30.5)	4,356 (25.5)	2.33 (1.60–3.40)
> 30	100 (18.7)	2,614 (15.3)	2.38 (1.60–3.55)
Lifetime days of exposure^b^			
0–225	286 (53.5)	9,907 (58.1)	Reference
226–752	153 (28.7)	4,579 (26.9)	1.16 (0.95–1.41)
> 752	95 (17.8)	2,565 (15.0)	1.28 (1.01–1.63)
HPEE			
No	397 (74.3)	14,622 (85.8)	Reference
Yes	137 (25.7)	2,429 (14.2)	2.08 (1.70–2.53)
Diagnosed poisoning			
No	499 (93.4)	16,754 (98.3)	Reference
Yes	35 (6.6)	297 (1.7)	3.96 (2.76–5.68)

a Exposure to solvents (other than gasoline) and exposure to metals (lead solder, welding, electroplating fumes, lead, mercury, cadmium, and other metals) from the nonfarm job held the longest.

b Exposure time ≤ median of 225 days is reference group; 226–752 is median to 90th percentile; > 752 is > 90th percentile; individuals were categorized as experiencing an HPEE or diagnosed pesticide poisoning regardless of cumulative days of pesticide use.

**Table 2:** Multivariable logistic regression analysis [adjusted OR (95% CI)] of cumulative exposure levels and diagnosed depression adjusting for covariates in those without an HPEE, those without a pesticide poisoning, and those with neither. a

Variable	Model 1 (total sample)	Model 2 (no HPEE)	Model 3 (no poisoning)	Model 4 (no HPEE or poisoning)
State of residence				
Iowa	Reference	Reference	Reference	Reference
North Carolina	0.70 (0.56–0.87)	0.69 (0.54–0.88)	0.65 (0.52–0.81)	0.67 (0.52–0.85)
Age (years)				
< 40	Reference	Reference	Reference	Reference
40–49	1.64 (1.26–2.14)	1.49 (1.09–2.03)	1.61 (1.23–2.12)	1.49 (1.09–2.04)
50–59	1.65 (1.25–2.18)	1.48 (1.07–2.04)	1.58 (1.19–2.10)	1.49 (1.07–2.06)
> 59	0.91 (0.67–1.24)	0.86 (0.60–1.22)	0.84 (0.61–1.16)	0.81 (0.56–1.16)
High school education				
Yes	Reference	Reference	Reference	Reference
No	1.40 (1.03–1.91)	1.44 (1.02–2.02)	1.34 (0.97–1.86)	1.41 (0.99–2.00)
Married				
Yes	Reference	Reference	Reference	Reference
No	1.77 (1.40–2.25)	1.65 (1.25–2.18)	1.85 (1.46–2.36)	1.71 (1.30–2.26)
Visits to doctor in past year				
None	Reference	Reference	Reference	Reference
One	1.86 (1.41–2.46)	1.87 (1.36–2.58)	1.96 (1.47–2.62)	1.97 (1.42–2.75)
More than one	4.55 (3.53–5.86)	4.73 (3.54–6.32)	4.83 (3.72–6.28)	5.03 (3.73–6.78)
Alcohol use in past year				
Never	1.48 (1.15–1.91)	1.56 (1.16–2.10)	1.46 (1.12–1.90)	1.55 (1.15–2.09)
< 3 per month	1.35 (1.06–1.72)	1.39 (1.04–1.85)	1.37 (1.07–1.76)	1.39 (1.04–1.85)
1–4 per week	Reference	Reference	Reference	Reference
Every/almost every day	1.29 (0.88–1.91)	1.54 (0.99–2.41)	1.34 (0.90–1.99)	1.52 (0.97–2.39)
Cigarette smoking				
Never smoker	Reference	Reference	Reference	Reference
Past smoker	1.30 (1.06–1.59)	1.24 (0.99–1.56)	1.32 (1.07–1.62)	1.25 (0.99–1.58)
Current smoker	1.34 (1.01–1.77)	1.13 (0.80–1.58)	1.36 (1.02–1.81)	1.12 (0.79–1.58)
Farms currently having no crops or animals				
No	Reference	Reference	Reference	Reference
Yes	2.51 (1.28–4.92)	2.18 (0.99–4.81)	2.76 (1.41–5.42)	2.33 (1.06–5.15)
Ever work job off farm				
No	Reference	Reference	Reference	Reference
Yes	1.13 (0.92–1.39)	1.22 (0.96–1.55)	1.23 (0.99–1.53)	1.23 (0.96–1.56)
Solvent exposure				
No	Reference	Reference	Reference	Reference
Yes	1.26 (1.01–1.59)	1.38 (1.06–1.79)	1.28 (1.02–1.62)	1.38 (1.06–1.81)
Lifetime days of pesticide exposure^b^				
0–225	Reference	Reference	Reference	Reference
226–752	1.07 (0.87–1.31)	1.21 (0.96–1.54)	1.15 (0.93–1.43)	1.21 (0.95–1.55)
> 752	1.11 (0.87–1.42)	1.51 (1.14–1.99)	1.30 (1.01–1.66)	1.54 (1.16–2.04)
HPEE				
No	Reference	NA	NA	NA
Yes	1.65 (1.33–2.05)			
Diagnosed poisoning				
No	Reference	NA	NA	NA
Yes	2.57 (1.74–3.79)			

NA, not analyzed. Model 1: cases = 534, controls = 17,051; model 2: cases = 397, controls = 14,622; model 3: cases = 499, controls = 16,754; model 4: cases = 385, controls = 14,530.

a Data from AHS, 1993–1997 (Alavanja et al. 1996).

b Exposure time ≤ median of 225 days is reference group; 226 to 752 is median to 90th percentile; > 752 is above the 90th percentile.

**Table 3:** Multivariable logistic regression analysis [OR (95% CI)] of exposure levels and diagnosed depression adjusting for demographic and farm characteristics^a^ comparing Iowa (*n* = 12,250) and North Carolina (*n* = 5,335) farmer applicators.^b^

Variable	Iowa	North Carolina
Lifetime days of pesticide exposure^c^		
0–225 days	Reference	Reference
226–752 days	1.07 (0.84–1.36)	1.07 (0.70–1.63)
> 752 days	0.98 (0.72–1.35)	1.35 (0.89–2.05)
HPEE		
No	Reference	Reference
Yes	1.72 (1.35–2.20)	1.39 (0.86–2.24)
Diagnosed poisoning		
No	Reference	Reference
Yes	2.36 (1.47–3.80)	3.04 (1.51–6.12)

For Iowa, cases = 385 and controls = 11,865; for North Carolina, cases = 149 and controls = 5,186.

a Adjusted by age, doctor visits, marital status, solvent exposure, alcohol, smoking, education, currently having no crops or animals and working a job off the farm.

b AHS, 1993–1997 (Alavanja et al. 1996).

c Exposure time ≤ median of 225 days is the reference group; 226–752 is median to 90th percentile; > 752 is above the 90th percentile.

**Table 4:** Unadjusted and adjusted ORs (95% CIs) from logistic regression models of diagnosed depression for specific classes of pesticides in 17,585 male farmer applicators.^a^,^b^

Pesticide class	Cases [no. (%)]	Controls [no. (%)]	Unadjusted OR (95% CI)	Adjusted OR (95% CI)
Ever used herbicides				
No	4 (0.7)	262 (1.5)	Reference	Reference
Yes	530 (99.3)	16,789 (98.5)	2.07 (0.77–5.57)	2.05 (0.76–5.54)
Ever used insecticides				
No	19 (3.6)	1,149 (6.7)	Reference	Reference
Yes	515 (96.4)	15,902 (93.3)	1.96 (1.23–3.11)	2.05 (1.29–3.27)
Ever used OPs				
No	38 (7.1)	1,969 (11.5)	Reference	Reference
Yes	496 (92.9)	15,082 (88.5)	1.70 (1.22–2.38)	1.78 (1.27–2.50)
Ever used carbamates				
No	219 (41.0)	7,329 (43.0)	Reference	Reference
Yes	315 (59.0)	9,722 (57.0)	1.08 (0.91–1.29)	1.10 (0.91–1.32)
Ever used organochlorines				
No	265 (49.6)	9,893 (58.0)	Reference	Reference
Yes	269 (50.4)	7,158 (42.0)	1.40 (1.18–1.67)	1.32 (1.09–1.59)
Ever used fungicides				
No	349 (65.4)	11,510 (67.5)	Reference	Reference
Yes	185 (34.6)	5,541 (32.5)	1.10 (0.92–1.32)	1.24 (1.01–1.53)
Ever used fumigants				
No	404 (75.7)	13,495 (79.1)	Reference	Reference
Yes	130 (24.3)	3,556 (20.9)	1.22 (1.00–1.49)	1.35 (1.07–1.69)

a Adjusted by state of residence, marital status, age, solvent exposure, alcohol, smoking, education, working a job off the farm, and having no crops or animals.

b Data from AHS, 1993–1997 (Alavanja et al. 1996).
